# Bloodstream and endovascular infections due to *Abiotrophia defectiva *and *Granulicatella *species

**DOI:** 10.1186/1471-2334-6-9

**Published:** 2006-01-20

**Authors:** Laurence Senn, José M Entenza, Gilbert Greub, Katia Jaton, Aline Wenger, Jacques Bille, Thierry Calandra, Guy Prod'hom

**Affiliations:** 1 Infectious Diseases Service, University Hospital, Lausanne, Switzerland; 2 Institute of Microbiology, University Hospital, Bugnon 46, 1011 Lausanne CHUV, Switzerland

## Abstract

**Background:**

*Abiotrophia *and *Granulicatella *species, previously referred to as nutritionally variant streptococci (NVS), are significant causative agents of endocarditis and bacteraemia. In this study, we reviewed the clinical manifestations of infections due to *A. defectiva *and *Granulicatella *species that occurred at our institution between 1998 and 2004.

**Methods:**

The analysis included all strains of NVS that were isolated from blood cultures or vascular graft specimens. All strains were identified by 16S rRNA sequence analysis. Patients' medical charts were reviewed for each case of infection.

**Results:**

Eleven strains of NVS were isolated during the 6-year period. Identification of the strains by 16S rRNA showed 2 genogroups: *Abiotrophia defectiva *(3) and *Granulicatella adiacens *(6) or *"para-adiacens" *(2). The three *A. defectiva *strains were isolated from immunocompetent patients with endovascular infections, whereas 7 of 8 *Granulicatella *spp. strains were isolated from immunosuppressed patients, mainly febrile neutropenic patients. We report the first case of *"G. para-adiacens" *bacteraemia in the setting of febrile neutropenia.

**Conclusion:**

We propose that *Granulicatella *spp. be considered as a possible agent of bacteraemia in neutropenic patients.

## Background

Nutritionally variant streptococci (NVS), first described in 1961 by Frenkel and Hirsch [1], were classified on the basis of growth characteristics such as nutrient requirements (pyridoxal) and presence of satellitism. In 1989, based on DNA-DNA hybridisation, Bouvet et al. showed that NVS could be divided in two groups, *Streptococcus defectivus *and *Streptococcus adiacens *[2]. In 1995, based on the genetic and phylogenetic analysis of the 16S rRNA sequences, the genus *Abiotrophia *and the two species *A. defectiva and A. adiacens *were proposed by Kawamura [3]. In 1998, Roggenkamp et al. proposed the new species *A. elegans *[4], and in 1999, Lawson et al. proposed the species *A. balaenopterae *[5]. Based on 16S rRNA heterogeneity and phenotypic differences, Kanamoto et al. proposed an additional species "*Abiotrophia para-adiacens" *[6]. In 2000, *A. adiacens, A. balaenopterae and A. elegans *were reclassified in the new genus *Granulicatella *by Collins and Lawson [7].

*Abiotrophia *and *Granulicatella *species form part of the normal flora of the oral cavity [8-10], the genitourinary tract, and the intestinal tract [11]. *G. adiacens *is isolated more frequently from oral specimens than other NVS [4,9,10]. Bacteraemia and endocarditis are the more frequently reported clinical infections due to *Abiotrophia *and *Granulicatella *species [12] and account for 4.3 to 6% of all "streptococcal" endocarditis [13]. Isolated cases of keratitis [14], endophthalmitis [15], central nervous system infections [16-20], sinusitis, otitis media, prostatitis, cholangitis, arthritis [21-23] and osteomyelitis [24,25] have also been reported. The high prevalence of beta-lactam and macrolide resistance among isolates of *Abiotrophia *and *Granulicatella *may pose a challenge to treat invasive infections [26-28].

In this study, we reviewed the clinical manifestations of infections due to *A. defectiva *and *Granulicatella *species that occurred at our institution over a 6-year period.

## Methods

### Bacterial strains

The analysis included all strains of NVS that were isolated from blood cultures or vascular graft specimens from patients admitted to our 800-bed University Hospital from January 1998 to December 2004. The automated blood culture system used in the microbiology laboratory during the study period was the Bactec 9240 (Becton Dickinson, Sparks, Md.) with the Plus aerobic/F and Lytic anaerobic/F vials (Becton Dickinson). The strains were identified to the species level using the Rapid ID32 STREPT system (Bio Mérieux SA, Marcy-l'Etoile, France).

### 16S rRNA gene sequencing

**Figure 1:**
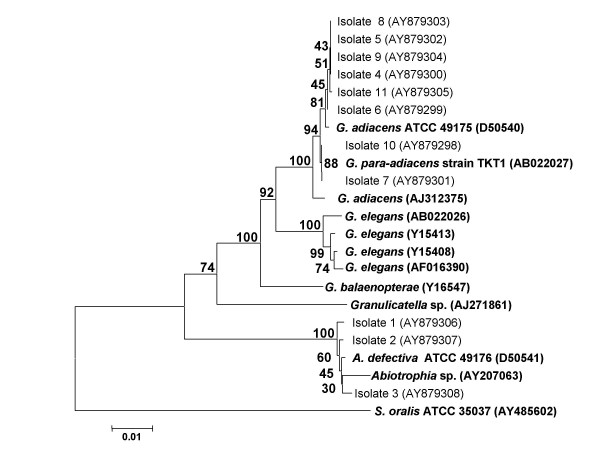
**Phylogenetic analysis of the strains**. Phylogenetic tree showing the affiliation of 3 isolates to *A. defectiva*, 6 isolates to *G. adiacens *and 2 isolates to "*G. para-adiacens*". The tree was inferred from 1315 base pairs 16S rRNA sequence data by the neighbour-joining method using the Kimura-corrected p-distance. *Streptococcus oralis *was used as outgroup. Genbank accession numbers are shown in parentheses.

All strains were also identified by 16S rRNA sequence analysis. DNA was extracted with the MagNA Pure LC DNA isolation Kit I (Roche Diagnostics, Mannheim, Germany) according to the instructions of the manufacturer. Polymerase chain reaction (PCR) amplification of the 16S RNA gene was performed with primers fD1 and rP2 [29] and Taq DNA polymerase (Gibco BRL, Life Technologies) followed by electrophoresis of the PCR products on ethidium bromide-stained 1% agarose gel. PCR products were purified using the QIAquick PCR purification kit (Qiagen, Courtaboeuf, France). Sequencing was performed by using the dRhodamine Terminator Cycle Sequencing Ready Reaction kit with one of six different primers and AmpliTaq DNA (Perkin-Elmer Biosystems, Warrington, England) with a 3100 ABI Prism automated sequencer (Applied Biosystems, Courtaboeuf, France). Sequences derived from each primer were aligned and combined into a single 16S rRNA sequence by using Contig Express, a component of the Vector NTI suite 9.0 (Informax, Frederick, MD). Each sequence was compared with all eubacterial 16S rRNA sequences available in the GenBank database by using the BLASTN 2.2.2 program available on the National Center for Biotechnology Information website [30,31]. The 16S rRNA sequences of *Abiotrophia *and *Granulicatella *isolates were aligned with those of other members of the genus *Abiotrophia *and *Granulicatella *by using the CLUSTWAL W program supported by the DDBJ website [32]. Sequences were edited by removal of the longer 5' and 3' ends so that their lengths matched that of the shortest sequence and then analysed by neighbour-joining, parsimony and minimum evolution methods (Kimura's correction, pairwise deletion option) using the Mega 2.1 software [33]. GenBank accession numbers are shown in Figure 1.

### Antimicrobial susceptibility testing

The minimal inhibitory concentrations (MIC) of penicillin, ceftriaxone, meropenem, clarithromycin, erythromycin, quinupristin/dalfopristin, levofloxacin, vancomycin and teicoplanin were determined for each isolate by the E-test method (AB Biodisk, Solna, Sweden), using Brucella agar supplemented with haemin, vitamin K1, cystein and 5% sheep blood as test medium (BA). For vancomycin, an E-test was also performed on Mueller-Hinton agar with 5% sheep blood (BMH) and 1% vitox defined supplement (Oxoid, Basel, Switzerland). Antimicrobial susceptibilities were interpreted according to the guidelines established by the CLSI for Streptococcus spp. other than *Streptococcus pneumoniae *[34].

### Clinical data

The patients' medical charts were reviewed and clinical characteristics (age, sex, clinical diagnosis, underlying conditions, predisposing factors, antibiotic treatment and outcome) were recorded for each case of infection due to NVS. Neutropenia was defined as a neutrophil count of <500 cells/mm^3^.

### Ethics

The design of this study was in accordance with the ethical standards of our hospital ethics committee. Because the study was retrospective, informed consent was not required.

## Results

During the six-year study period, ten patients had positive blood cultures for NVS and one had a positive culture of a vascular graft fragment. All but one positive blood culture were detected within the first twenty-four hours of incubation. The strains grew equally from both vials. All strains showed satellitism around streaks of *Staphylococcus aureus*. Seven of 11 strains were identified successfully to the species level using the Rapid ID32 STREPT system.

Based on their 16S rRNA sequences, 3 strains were identified as *A. defectiva *(isolates 1–3) and 8 as *Granulicatella *spp. (isolates 4–11). Isolates 1, 2 and 3 exhibited 99.6 to 99.7% 16S rRNA homology with *A. defectiva *strain ATCC 49176 and 92.1 to 92.4% 16S rRNA homology with *G. adiacens *strain ATCC 49175. Isolates 4 to 11 exhibited 92.2 to 92.5% 16S rRNA homology with *A. defectiva *strain ATCC 49176 and 99.8% 16S rRNA homology with *G. adiacens *strain ATCC 49175. Two strains, isolates 7 and 10, exhibited 100% 16S rRNA homology with *"G. para-adiacens" *strain TKT1 [6]. The phylogenetic tree shown in Fig. 1 demonstrates the affiliation of strains 1,2 and 3 to the genus *Abiotrophia*. Phylogenetic analyses confirmed affiliation of strains 4, 5, 6, 8, 9 and 11 to *G. adiacens *and strains 7 and 10 to *"G. para-adiacens" *(Fig. 1). Strains 7 and 10 clustered with *"G. para-adiacens" *strain TKT1 with bootstraps of 94%, 84% and 80% in neighbour-joining, parsimony and minimum evolution analysis, supporting the node separating them from their closest neighbour *G. adiacens*.

According to CLSI interpretive criteria, the 3 *A. defectiva *strains were susceptible to all the antibiotics tested except 1 strain with a reduced susceptibly to penicillin (MIC 0.25 μg/ml). Two *Granulicatella *spp. showed resistance to penicillin (MIC 4 and 8 μg/ml, respectively), and intermediate susceptibility (MIC 2 μg/ml) or resistance (MIC 8 μg/ml) to ceftriaxone. One *Granulicatella *spp. was intermediately susceptible to penicillin (MIC 0.25 μg/ml) but sensitive to other beta-lactams. Two other strains were intermediately susceptible to ceftriaxone alone (MIC 2 μg/ml). All *Granulicatella *spp. strains showed vancomycin MICs of 1.5 to 2 μg/ml and teicoplanin MICs of <0.25 μg/ml with BA. MICs to vancomycin were lower when tested on BMH (MIC 0.5 to 1 μg/ml). All *Granulicatella *spp. strains were susceptible to meropenem, clarithromycin, erythromycin, quinupristin/dalfopristin, and levofloxacin.

The epidemiological and clinical characteristics of the patients are shown in Additional file 1. Two patients with *A. defectiva *infections had endocarditis, one of which also had sacroileitis. The third patient had polymicrobial (*A. defectiva *plus *Escherichia coli*) vascular graft infection related to an aorto-enteric fistula.

Seven of 8 patients with bacteraemia due to *Granulicatella *spp. were immunosuppressed. Underlying conditions included haematological malignancies (5), lung cancer (1) and advanced metastatic oesophageal cancer (1). Three patients had polymicrobial infections (*G. adiacens *plus *Clostridium sordellii, Staphylococcus epidermidis *or *Lactobacillus rhamnosus*). Six patients presented with febrile chemotherapy-induced neutropenia and mucositis, including one patient with possible infection of a catheter and one with possible endocarditis. One of the six patients with neutropenia died 10 days after bacteraemia from gastrointestinal bleeding in the setting of refractory thrombopenia. The other 5 neutropenic patients clinically improved clinically with intravenous antibiotic therapy. The 3 patients with primary bacteraemia due to *Granulicatella *spp. with intermediate susceptibility or resistance to penicillin were successfully treated (cases 7, 10 and 11).

## Discussion

*A. defectiva *and *Granulicatella *spp. are now considered as two distinct genera based on 16S rRNA tree topology and sequence divergence considerations [7]. The review of these clinical cases suggests that each species is associated with a distinct clinical presentation: *A. defectiva *infections were seen in immunocompetent patients with endovascular infections, whereas 7 of 8 *Granulicatella *spp. bacteraemia occurred in immunosuppressed, mainly febrile neutropenic patients. To date, no cases of *A. defectiva *and only five cases of *Granulicatella *spp. bacteraemia in neutropenic patients have been reported. Pierard et al. described one case of *G. adiacens *bacteraemia among 62 cases of streptococcal bacteraemia in neutropenic patients [35]. In a small series published by Woo et al., three cases of *G. adiacens *bacteraemia were associated with febrile neutropenia in cancer patients [36]. Finally, one case of *G. elegans *bacteraemia was reported by Murray et al. in a febrile neutropenic cancer patient [37]. We report here the first case of *"G. para-adiacens" *infection in the setting of febrile neutropenia.

One and two of 8 *Granulicatella *spp. strains, respectively, showed reduced susceptibility and resistance to penicillin. This rate of resistance is similar to the prevalence of penicillin resistance recently described [26,28]. This suggests that antimicrobial susceptibility testing should be systematically done in order to select appropriate antimicrobial therapy. In severely ill patients or those with a suboptimal response to initial therapy with beta-lactam antibiotics, treatment with vancomycin should be considered. We did not observed therapeutic failures; the fatal outcome in two cases was not attributed to the infection.

All *Abiotrophia *and *Granulicatella *strains were susceptible to vancomycin. However, depending on the culture medium used for E-test method, we observed discrepant results. Overestimation of vancomycin E-test values have previously been reported for *Strepococci *spp., when compared to values obtained with broth or agar dilution methods [38-40]. Vancomycin E-test values should thus be interpreted with caution.

As observed in this series, *Granulicatella *spp. bacteraemia may occur in the setting of chemotherapy-associated mucositis and neutropenia. Oro-intestinal colonisation by *Granulicatella *spp. and subsequent mucositis may represent predisposing factors for bacteraemia in neutropenic patients, as it is well documented for viridans streptococci [41,42]. The absence of cases of bacteraemia due to *A. defectiva *in neutropenic patients could reflect a lower frequency of oral colonisation by this species in comparison to *Granulicatella *spp. In one study, the rates of oral colonisation in healthy students were 11.8% and 87.1% for *A. defectiva *and *G. adiacens*, respectively [9].

## Conclusion

We report six cases of bacteraemia due to *Granulicatella *spp. in febrile neutropenic patients. Chemotherapy-induced neutropenia and oral mucositis may represent predisposing factors. *Granulicatella *spp. should be considered as a possible agent of bacteraemia in neutropenic cancer patients.

## Competing interests

The author(s) declare that they have no competing interests.

## Authors' contributions

LS collected the clinical data, carried out the 16S rRNA sequencing and wrote the draft of the manuscript. AW, JME and GP did the microbiological studies. KJ and GG participated in the 16S rRNA sequencing. JME, GG, JB, TC and GP provided input into subsequent drafts of this manuscript. All authors read and approved the final version of manuscript.

## Pre-publication history

The pre-publication history for this paper can be accessed here:



## Supplementary Material

Additional File 1 **Clinical characteristics of 11 patients with bloodstream and endovascular infections due to *Abiotrophia defectiva *and *Granulicatella *spp**. This table summarizes patients' age, sex, clinical diagnosis, species identification, Genbank accession number, underlying conditions, predisposing factors, antimicrobial therapy and outcome. Click here for file

## References

[B1] FRENKEL A, HIRSCH W (1961). Spontaneous development of L forms of streptococci requiring secretions of other bacteria or sulphydryl compounds for normal growth. Nature.

[B2] Bouvet A, Grimont F, Grimont PAD (1989). Streptococcus defectivus sp. nov and Streptococcus adjacens sp. nov., nutritionally variant streptococci from human clinical specimens.. International Journal of Systematic Bacteriology.

[B3] Kawamura Y, Hou XG, Sultana F, Liu S, Yamamoto H, Ezaki T (1995). Transfer of Streptococcus adjacens and Streptococcus defectivus to Abiotrophia gen. nov. as Abiotrophia adiacens comb. nov. and Abiotrophia defectiva comb. nov., respectively. Int J Syst Bacteriol.

[B4] Roggenkamp A, bele-Horn M, Trebesius KH, Tretter U, Autenrieth IB, Heesemann J (1998). Abiotrophia elegans sp. nov., a possible pathogen in patients with culture-negative endocarditis. J Clin Microbiol.

[B5] Lawson PA, Foster G, Falsen E, Sjoden B, Collins MD (1999). Abiotrophia balaenopterae sp. nov., isolated from the minke whale (Balaenoptera acutorostrata). Int J Syst Bacteriol.

[B6] Kanamoto T, Sato S, Inoue M (2000). Genetic heterogeneities and phenotypic characteristics of strains of the genus Abiotrophia and proposal of Abiotrophia para-adiacens sp. nov. J Clin Microbiol.

[B7] Collins MD, Lawson PA (2000). The genus Abiotrophia (Kawamura et al.) is not monophyletic: proposal of Granulicatella gen. nov., Granulicatella adiacens comb. nov., Granulicatella elegans comb. nov. and Granulicatella balaenopterae comb. nov. Int J Syst Evol Microbiol.

[B8] Mikkelsen L, Theilade E, Poulsen K (2000). Abiotrophia species in early dental plaque. Oral Microbiol Immunol.

[B9] Ohara-Nemoto Y, Tajika S, Sasaki M, Kaneko M (1997). Identification of Abiotrophia adiacens and Abiotrophia defectiva by 16S rRNA gene PCR and restriction fragment length polymorphism analysis. J Clin Microbiol.

[B10] Sato S, Kanamoto T, Inoue M (1999). Abiotrophia elegans strains comprise 8% of the nutritionally variant streptococci isolated from the human mouth. J Clin Microbiol.

[B11] Ruoff KL (1991). Nutritionally variant streptococci. Clin Microbiol Rev.

[B12] Christensen JJ, Facklam RR (2001). Granulicatella and Abiotrophia species from human clinical specimens. J Clin Microbiol.

[B13] Brouqui P, Raoult D (2001). Endocarditis due to rare and fastidious bacteria. Clin Microbiol Rev.

[B14] Keay L, Harmis N, Corrigan K, Sweeney D, Willcox M (2000). Infiltrative keratitis associated with extended wear of hydrogel lenses and Abiotrophia defectiva. Cornea.

[B15] Namdari H, Kintner K, Jackson BA, Namdari S, Hughes JL, Peairs RR, Savage DJ (1999). Abiotrophia species as a cause of endophthalmitis following cataract extraction. J Clin Microbiol.

[B16] Michelow IC, McCracken GHJ, Luckett PM, Krisher K (2000). Abiotrophia spp. brain abscess in a child with Down's syndrome. Pediatr Infect Dis J.

[B17] Schlegel L, Merlet C, Laroche JM, Fremaux A, Geslin P (1999). Iatrogenic meningitis due to Abiotrophia defectiva after myelography. Clin Infect Dis.

[B18] Biermann C, Fries G, Jehnichen P, Bhakdi S, Husmann M (1999). Isolation of Abiotrophia adiacens from a brain abscess which developed in a patient after neurosurgery. J Clin Microbiol.

[B19] Zenone T, Durand DV (2004). Brain abscesses caused by Abiotrophia defectiva: complication of immunosuppressive therapy in a patient with connective-tissue disease. Scand J Infect Dis.

[B20] Cerceo E, Christie JD, Nachamkin I, Lautenbach E (2004). Central nervous system infections due to Abiotrophia and Granulicatella species: an emerging challenge?. Diagn Microbiol Infect Dis.

[B21] Hepburn MJ, Fraser SL, Rennie TA, Singleton CM, Delgado BJ (2003). Septic arthritis caused by Granulicatella adiacens: diagnosis by inoculation of synovial fluid into blood culture bottles. Rheumatol Int.

[B22] Riede U, Graber P, Ochsner PE (2004). Granulicatella (Abiotrophia) adiacens infection associated with a total knee arthroplasty. Scand J Infect Dis.

[B23] Wilhelm N, Sire S, Le CA, Loubinoux J, Beljerd M, Bouvet A (2005). First case of multiple discitis and sacroiliitis due to Abiotrophia defectiva. Eur J Clin Microbiol Infect Dis.

[B24] Heath CH, Bowen SF, McCarthy JS, Dwyer B (1998). Vertebral osteomyelitis and discitis associated with Abiotrophia adiacens (nutritionally variant streptococcus) infection. Aust N Z J Med.

[B25] Rosenthal O, Woywodt A, Kirschner P, Haller H (2002). Vertebral osteomyelitis and endocarditis of a pacemaker lead due to Granulicatella (Abiotrophia) adiacens. Infection.

[B26] Tuohy MJ, Procop GW, Washington JA (2000). Antimicrobial susceptibility of Abiotrophia adiacens and Abiotrophia defectiva. Diagn Microbiol Infect Dis.

[B27] Zheng X, Freeman AF, Villafranca J, Shortridge D, Beyer J, Kabat W, Dembkowski K, Shulman ST (2004). Antimicrobial susceptibilities of invasive pediatric Abiotrophia and Granulicatella isolates. J Clin Microbiol.

[B28] Liao CH, Teng LJ, Hsueh PR, Chen YC, Huang LM, Chang SC, Ho SW (2004). Nutritionally variant streptococcal infections at a University Hospital in Taiwan: disease emergence and high prevalence of beta-lactam and macrolide resistance. Clin Infect Dis.

[B29] Weisburg WG, Barns SM, Pelletier DA, Lane DJ (1991). 16S ribosomal DNA amplification for phylogenetic study. J Bacteriol.

[B30] Altschul SF, Madden TL, Schaffer AA, Zhang J, Zhang Z, Miller W, Lipman DJ (1997). Gapped BLAST and PSI-BLAST: a new generation of protein database search programs. Nucleic Acids Res.

[B31] [http://www.ncbi.nlm.nih.gov].

[B32] [http://www.ddbj.nig.ac.jp].

[B33] Kumar S, Tamura K, Jakobsen IB, Nei M (2001). MEGA2: molecular evolutionary genetics analysis software. Bioinformatics.

[B34] Institute CS (2005). Performance standards for antimicrobial susceptibility testing: fifteenth informational supplement. Clinical and laboratory Standards Institute, Wayne, Pa.

[B35] Pierard D, de MA, Lauwers S (1994). Antibiotic susceptibility of streptococci isolated from blood from neutropenic patients. Pathol Biol (Paris).

[B36] Woo PC, Fung AM, Lau SK, Chan BY, Chiu SK, Teng JL, Que TL, Yung RW, Yuen KY (2003). Granulicatella adiacens and Abiotrophia defectiva bacteraemia characterized by 16S rRNA gene sequencing. J Med Microbiol.

[B37] Murray CK, Walter EA, Crawford S, McElmeel ML, Jorgensen JH (2001). Abiotrophia bacteremia in a patient with neutropenic fever and antimicrobial susceptibility testing of Abiotrophia isolates. Clin Infect Dis.

[B38] Smith A, Jackson MS, Kennedy H (2004). Antimicrobial susceptibility of viridans group streptococcal blood isolates to eight antimicrobial agents. Scand J Infect Dis.

[B39] Rosser SJ, Alfa MJ, Hoban S, Kennedy J, Harding GK (1999). E test versus agar dilution for antimicrobial susceptibility testing of viridans group streptococci. J Clin Microbiol.

[B40] Hashemi FB, Schutze GE, Mason EOJ (1996). Discrepancies between results by E-test and standard microbroth dilution testing of Streptococcus pneumoniae for susceptibility to vancomycin. J Clin Microbiol.

[B41] Marron A, Carratala J, Gonzalez-Barca E, Fernandez-Sevilla A, Alcaide F, Gudiol F (2000). Serious complications of bacteremia caused by Viridans streptococci in neutropenic patients with cancer. Clin Infect Dis.

[B42] Richard P, Amador DV, Moreau P, Milpied N, Felice MP, Daeschler T, Harousseau JL, Richet H (1995). Viridans streptococcal bacteraemia in patients with neutropenia. Lancet.

