# Supplementary material for: Bloodstream and endovascular infections due to Abiotrophia defectiva and Granulicatella species
Source: BMC Infect Dis. 2006 Jan 20;6:9. doi: 10.1186/1471-2334-6-9 (PMC1360077; doi:10.1186/1471-2334-6-9)
Supplement: Additional File 1 — Clinical characteristics of 11 patients with bloodstream and endovascular infections due to Abiotrophia defectiva and Granulicatella spp. This table summarizes patients' age, sex, clinical diagnosis, species identification, Genbank accession number, underlying conditions, predisposing factors, antimicrobial therapy and outcome. [file 1471-2334-6-9-S1.doc]

**Table 1: Clinical characteristics of 11 patients with bloodstream and endovascular infections due to *Abiotrophia defectiva* and *Granulicatella* spp.**

| **Case no.** | **Sex/Age** | **Clinical diagnosis** | **16 SrRNA identification** | **Genbank accession number** | **Mono-/ polymicrobial bacteraemia** | **Underlying conditions** | **Predisposing factors** | **Antibimicrobial therapy** | **Outcome** |
| --- | --- | --- | --- | --- | --- | --- | --- | --- | --- |
| 1 | F/58 | Mitral endocarditis and sacroileitis | A. defectiva | AY879306 | Monomicrobial | Rheumatic fever in childhood | None | Amoxicillin/clavulanate and amikacin for 1w, then ceftriaxone for 3w | Cured |
| 2 | M/55 | Aortic endocarditis | *A. defectiva* | AY879307 | Monomicrobial | None | None | Ceftriaxone for 6w and amikacin for 4w | Cured |
| 3 | M/67 | Aortic graft infection | *A. defectiva* | AY879308 | Polymicrobial  (+ *Escherichia coli*) | Aorto-femoral vascular graft | Aortoenteric fistula | Amoxicillin/clavulanate for 2w, then ciprofloxacin for > 24w | No long term follow-up  (vascular graft not removed) |
| 4 | F/58 | Primary bacteraemia | *G. adiacens* | AY879300 | Monomicrobial | Chronic lymphocytic leukaemia | Neutropenia | Cefepime for 2d, then piperacillin/tazobactam for 3d , then ceftriaxone and gentamicin for 10d | Death  (gastrointestinal bleeding/ refractory thrombopenia) |
| 5 | F/56 | Primary bacteraemia | *G. adiacens* | AY879302 | Polymicrobial  (+ *Staphylococcus epidermidis*) | Acute lymphoblastic leukaemia | Neutropenia  Oral mucositis | Cefepime and vancomycin for 2d, then imipenem for 2w | Cured |
| 6 | M/62 | Primary bacteraemia | *G. adiacens* | AY879299 | Polymicrobial  (+ *Clostridium sordellii*) | Small cell lung cancer | Neutropenia Autologous BMT  Oral and intestinal mucositis | Cefepime for 4d, then amoxicillin for 10d | Cured |
| 7 | M/1 | Primary bacteraemia | *“G. para-adiacens”* | AY879301 | Monomicrobial | Acute myeloid leukaemia | Neutropenia  Oral mucositis | Ceftriaxone and amikacin for 5d, then vancomycin for 10d | Cured |
| 8 | F/56 | Primary bacteraemia | *G. adiacens* | AY879303 | Monomicrobial | Multiple myeloma | Neutropenia Autologous BMT  Oral mucositis | Cefepime and vancomycin for 5d, then amoxicillin for 10d | Cured |
| 9 | M/62 | Primary bacteraemia | *G. adiacens* | AY879304 | Polymicrobial  (+ *Lactobacillus rhamnosus*) | Metastatic oesophageal cancer | Ulcerous oesophageal lesions | Amoxicilline/clavulanate for 8d, then imipenem for 2d | Death  (advanced metastatic cancer) |
| 10 | F/35 | Primary bacteraemia | *“G. para-adiacens”* | AY879298 | Monomicrobial | Hypertrophic cardiomyopathy | Cardiac allograft | Amoxicilline/clavulanate for 4d, then cefepime for 2d, then ceftriaxone for 2w | Cured |
| 11 | F/39 | Primary bacteraemia | *G. adiacens* | AY879305 | Monomicrobial | Lymphoma | Neutropenia  Autologous BMT | Cefepime and vancomycin for 1w, gentamicin for 4d, then imipenem for 1w | Cured |

M, male; F, female; BMT, Bone Marrow Transplant; d, day; w, week; m, month
